# ANTICIPATED SUICIDAL AND DEATH IDEATION IN RESPONSE TO AN IMAGINED DEMENTIA DIAGNOSIS

**DOI:** 10.1093/geroni/igac059.2824

**Published:** 2022-12-20

**Authors:** Molly Maxfield, Allie Peckham, Dara James, Laura Lathrop, Amy Fiske

**Affiliations:** Arizona State University, Phoenix, Arizona, United States; Arizona State University, Phoenix, Arizona, United States; University of South Alabama, Mobile, Alabama, United States; University of Denver, Denver, Colorado, United States; West Virginia University, Morgantown, West Virginia, United States

## Abstract

Alzheimer’s disease and related dementias are prevalent, incurable, and highly impactful diagnoses. Dementias are therefore feared diagnoses. Dementia-related anxiety (DRA) is anxiety about a current or future diagnosis of dementia and the associated complex symptoms. In a mixed methods study, semi-structured interviews were conducted to identify causes of DRA and revealed that numerous adults anticipated suicidal or death ideation if diagnosed with dementia. Fifty cognitively healthy, community-dwelling adults aged 58 to 89 (M = 70.92, SD = 6.08; 64% female) were recruited from a university participant registry and Memory Clinic. Among participants endorsing anticipated suicidal or death ideation, responses ranged from active plans, including interest in physician-assisted suicide, to more passive wishes to hasten death rather than continue to live with dementia. Within reports of both anticipated suicidal and death ideation, subthemes emerged, including the concern about becoming a burden to others in more advanced stages of dementia, the devaluation of life or the self with dementia, and the desire for (and anticipated thwarting of) control and independence. Statements of anticipated suicidal and death ideation were contingent on a future dementia diagnosis and may reflect errors in affective forecasting. Nevertheless, given the prevalence of dementias and older adults’ elevated rates of suicide, the intersection of these two public health issues warrants greater attention and further investigation.

